# Participation in nighttime activities in the genesis of depression in public school teachers from the State of Pernambuco, Brazil

**DOI:** 10.1590/S1980-57642012DN06040013

**Published:** 2012

**Authors:** Francisca Maria da Silva Correia, Rosângela Nieto de Albuquerque, Hugo André de Lima Martins, Luciano da Fonseca Lins, Murilo Duarte Costa Lima, José Marcos da Silva Dias, Cícera Maria da Silva, Allison José dos Santos, Leonardo Tárcito dos Santos, Valdenilson Ribeiro Ribas

**Affiliations:** 1 Master Student in Educational Psychology.; 2 Doctor in Neuropsychiatry. 3Master in Language Sciences.; 3 Master in Language Sciences.; 4 Doctor in Neuropsychiatry.; 5 Post-doctorate in Science Education.; 6 Doctor in Medicine.; 7 Expert in Educational Psychology.; 8 Graduate Student in Education.; 9 Expert in Educational Psychology.; 10 Masters Student in Organizational Ssychology.

**Keywords:** female teachers, excessive daytime sleepiness, depression

## Abstract

Teachers often undertake nighttime work involving exam corrections, projects and devising lesson plans in their homes. Many present excessive daytime sleepiness (EDS) and depression.

**Objective:**

The objective of this study was to evaluate EDS and depression in teachers from public schools.

**Methods:**

201 female teachers were evaluated in the district of Quipapá/PE, Brazil. Among the study sample, 38 working 1 shift (CONTROL 1), 40 working 2 shifts (CONTROL 2) and 123 working 3 shifts (WTeachers-3T). The subjects were submitted to evaluation by the Epworth Sleepiness Scale and Beck Depression Inventory (BDI).The EDS data were analyzed by the Kruskal-Wallis test with Dunn's multiple comparison, p<0.05and expressed in MEDIAN (MINIMUM - MAXIMUM) whereas the depression data were analyzed by the Chi-square test, with p<0.05, expressed in percentage.

**Results:**

WTeachers-3T presented excessive daytime sleepiness and higher rates of mild (24%) and moderate (37%) depression compared to controls - Control 1: mild (8%) and moderate (11%) - Control 2: mild (5%) and moderate (15%).

**Conclusion:**

This study found that teachers in the Quipapá municipality of Penambuco state working three shifts showed excessive daytime sleepiness and a higher percentage of mild and moderate depression compared to teachers working only one (1) or two (2) shifts.

## Abstract (pt)

Professores têm trabalhado muito no período noturno com correções de provas, trabalhos e elaborando planejamento de ensino em suas casas. Muitos estão apresentando sonolência excessiva diurna (SED) e depressão.

**Objetivo:**

O objetivo deste trabalho foi avaliar sonolência excessiva diurna e depressão em professoras da rede pública.

**Métodos:**

Foram avaliadas 201 professoras do município de Quipapá/PE, Brasil, sendo 38 que trabalham em 1 turno (CONTROLE 1), 40 que trabalham em 2 turnos (CONTROLE 2) e 123 que trabalham em 3 turnos (PROFas-3T). Os sujeitos foram avaliados pela Escala de Sonolência de Epworth e pelo Inventário de Depressão de Beck. Os dados referentes à SE foram analisados pelo teste Kruskal-Wallis com comparação múltipla em Dunn, p<0,05 e expressos em MEDIANA (MÍNIMO - MÁXIMO) e os dados referentes à depressão foram analisados pelo teste qui-quadrado, com p<0,05, expressos em percentuais.

**Resultados:**

PROFas-3T apresentam sonolência excessiva e apresentam maior percentual de depressão leve (24%) e moderada (37%) comparadas a seus controles - CONTROLE 1: leve (8%) e moderada (11%) - CONTROLE 2: leve (5%) e moderada (15%).

**Conclusão:**

Este estudo observou que professoras da rede municipal de Quipapá/PE que trabalham em 3 turnos apresentam sonolência excessiva e maior percentual de depressão leve e moderada comparadas às professoras que trabalham em 1 (um) ou em 2 (dois) turnos.

## INTRODUCTION

The World Health Organization (WHO) estimates that 450 million people in the world are suffering from some type of mental disorder. These diseases that can cause severe disability and stigmatization in the lives of these individuals and their families. Such individuals suffer when they become dependent on other people, often rendered incapable of participating in work and leisure activities. They cannot perform their responsibilities within the family and with friends and typically suffer discrimination by society. There are multiple impacts of mental and behavioral disorders in economic terms, but only part of this is measurable. However, the major effect can be highlighted as follows: need for social and health services, loss of a job, reduced productivity, impact on families, level of criminality and premature mortality.^1^

Among the numerous mental disorders, depression has been considered one of the ten main causes of disability in the world, limiting physical, personal and social functioning of affected individuals.^2^ However, few people affected seek or receive proper treatment, principally because of the process of stigma leading to them hiding the condition and seeking palliative treatment.^3^

The term depression, in its clinical context, does not refer to depressed mood, but to the syndrome characterized by mood changes and by a variety of somatic and neurovegetative disorders. When defining a diagnosis of depression, emotional or affective, physiological and behavior symptoms are taken into account.^4^

All human beings are familiar with emotions of happiness and sadness. Healthy individuals experience these emotions in a predictable way, usually in response to an external stimulus. These emotions are experienced by healthy individuals to some extent and for a period of time commensurate for the situation. Those individuals who suffer from depression however, experience a profound sadness apparently unrelated to external stimuli and for a long period.^5^ Sadness and depression may be associated with one another, but depression is a disease and not a feeling. In both cases the same internal anguish is evident, the same loss of interest in a dry world, empty, with the same isolation from other people, loneliness and feeling of emptiness.^6^

The latest report of the World Health Organization (WHO) presented Depression as a disease more commonly found in women, with an estimated prevalence of Depressive Episodes of 1.9% in males and 3.2% in females. Moreover, this report estimated that 5.8% of men and 9.5% of women experience a Depressive Episode over a period of 12 months. These prevalence rates vary among different populations.

Although depression may affect people in any phase of life, its incidence is higher during adolescence and in early adult life.^1^

Depression is essentially a disease that is manifested by recurrent Depressive Episodes. Each episode lasts from some months to some years, with a period of normality between them. In approximately 20% of cases, however, Depression follows a chronic course without remission, i.e., persists continuously (WHO), especially when there is no adequate treatment available.^7^

Some statistical data show that Depression affects from 15% to 20% of women and 5% to 10% of men. Approximately 2/3 of people with Depression do not undergo treatment and among patients that seek general practitioners, only 50% are correctly diagnosed.^8^

There are many professions that involve nighttime working, such as airport workers, health professionals, security guards and police officers among others. Although not shift work, evening activities in the teaching profession seem to be routine during resting hours, with many teachers performing exam corrections and devising lesson plans in their homes.^9^

Various external and internal elements that interact in maintaining the circadian cycle (from Latin: circa=around; dies=of the day) guide the capacity of the individual to adjust their cycle of sleep and wakefulness to remain in line with the night-day cycle of the Earth. Thus, the light and heat of the day, darkness, variations in light incidence throughout the day, clocks, sounds of the cities and animals (cock, birds, etc...) are all elements that condition us to maintain a pace of activity alternating with rest and interspersed with functions of food ingestion and elimination, all in synchrony with the circadian pattern. Experiments with human volunteers deprived of their natural environments and all these elements indicating the night-day cycle, show that the endogenous cycle in humans is around 25 hours, not necessarily obeying the 24 hours of the geological day. Such experimental environments were initially caves with camps improvised with artificial lighting, or more recently closed apartments isolated from any sound or external light, and devoid of clocks, TV or Internet that could give the individual clues as to the time of day.^10^

In this type of situation, it is observed that the subjects begin to organize their activities in such a way that their night sleep starts with a delay of one hour every day in relation to the previous day, completing a 25hour period, counting from the moment of awakening after the sustained sleep period, corresponding to the night sleep, until the next awakening. At the end of a period of 25 days, the subject returns to the time that the experiment began. This is called the free cycle.

Some people have difficulties synchronizing their cycle of rest-activity, wake-sleep with the geological and social cycle, even in the face of all the parameters of conditioning found in the environment. Thus, they have a period of irregular sleep, always tending to delay one hour per day in relation to the beginning of night sleep. This difficulty, when endogenous and not caused by poor sleep hygiene, has been classified as a disorder of the circadian cycle, called "different cycle of 24 hours".

From the endogenous perspective, the human organism exhibits complex cycles of hormonal secretion of neurotransmitters, as well as patterns of activity of certain brain centers, which are linked to the external synchronizers in order to allow a variation of the bio-rhythm of rest and activity, in agreement with the cir-cadian cycle of the Earth. One of the most important brain centers in this synchronization is the supraoptic nucleus in the anterior hypothalamus, which receives light impulses carried by the optic nerve, with light serving as one of the elements that controls the functioning of this center. The light stimuli also act on the pineal gland, which secretes melatonin, a neurohormone involved in the chronobiology of the sleep-wake cycle. The secretion of melatonin follows a programmed pattern, which is influenced by environmental light, with its peak in the early hours of the night and participating in the tendency of the individual to fall sleep. This peak is considered one of the "gates" of the entrance into a sleep state. Thus, if an individual forces the wakefulness state, struggling against sleep at this propitious time, they lose the entrance through this gate determined by the secretion peak of melatonin, subsequently having difficulties to fall sleep. Obviously, melatonin is not the only element determining this frequency of the wake-sleep cycle in humans, but is certainly recognized as one of the most important neurohormones.^10^

The secretion of some hormones and neurotrans-mitters is linked to the wake-sleep cycle, facilitating the wakefulness state or sleep state. Thus, in the first few hours of the morning, there is an increase in the secretion of the thyroid hormone, cortisol and insulin, which are facilitators of wakefulness, because of the increase in the metabolic rate for commencing activities of the day, or indirectly because of the increase of glycemia and use of glucose by cells.^11^

Although several causes of depression are known, this work sought to investigate and discuss, highlighting the interference caused by the performance of nighttime activities as one of the contributing factors in the etiology of depression among teachers.

Thus, the following questions emerge: Are Brazilian teachers indeed dedicating part of their nighttime rest period to work? If true, is this behavior affecting their sleep architecture? Are these professionals presenting depression? Does the nighttime working habit have its genesis in Brazilian society or is there interference from multiculturalism?

## METHODS

**Subjects.** A total of 201 teachers from public schools of Quipapa/PE were evaluated, comprising 38 professionals working 1 shift, 40 working 2 shifts and 123 working 3 shifts. The subjects were submitted to evaluations using the Epworth Sleepiness Scale and by a questionnaire with 21 groups of answers, each containing 4 (four) affirmative statements related to depression symptoms. When tallied at the end of the test, scores enable the identification of depression or otherwise, under the following classifications: absent - mild - moderate or severe (Beck Depression Inventory). The tests were performed in a room, under standard conditions, within a building with ceiling fans, at a temperature of 29º±2º C. Only female subjects that reported dedicating three days of the week to test corrections activities and schoolwork and sleeping only four hours were included whereas all male subjects were excluded given the small number of men.

**Groups.** The subjects were divided into three groups: female teachers working 1 shift (CONTROL 1), n=38; teachers working 2 shifts (CONTROL 2), n=40; and teachers working 3 shifts (WTeachers-3T), n=123.

**Evaluations.** This study was approved by the Research Ethics Committee of the Restoration Hospital, homologated on September 16, 2009.

Before data collection, all the evaluated subjects signed the Free and Informed Consent Form (FCCT).

Instruments used: *Epworth Sleepiness Scale (ESS)* - The Epworth Sleepiness Scale is a self-report questionnaire with eight questions, each describing in more detail the chance of dozing off in each one of them. These encompass different situations, all commonly found in everyday life.

The question posed is: how likely are you to doze off in the following situations: watching TV? - Reading? - Lying down in the afternoon? - In a public meeting? - Sitting after lunch? - Talking to someone? - In a car sitting for more than one hour?

The answers must be written in numerical form from 0 to 3, with the following meanings: 0 no chance of dozing, 1: slight chance of dozing, 2: moderate chance of dozing and 3: high chance of dozing.^12^

BDI is a self-report scale with 21 items, each having four alternatives, indicating increasing degrees of depression severity, with scores from 0 to 3. According to Beck & Steer (1993b)^13^ the estimated time for test completion is from 5 to 10 minutes for the self-report form and around 15 minutes for the version applied orally by an examiner, although for very obsessed patients this may take up to 30 minutes.

The items were selected based on observations and reports of most frequent symptoms and attitudes in psychiatric patients with depressive disorders and were not chosen on the basis of any particular theory of depression. The BDI items refer to:

[1] Sadness;
[2] Pessimism;
[3] Past failures;
[4] Loss of pleasure;
[5] Guilty feelings;
[6] Punishment feelings;
[7] Self-dislike;
[8] Self-criticalness;
[9] Suicidal ideation;
[10] Crying;
[11] Irritability;
[12] Social withdrawal;
[13] Indecisiveness;
[14] Body image change;
[15] Work difficulties;
[16] Insomnia;
[17] Fatigue;
[18] Loss of appetite;
[19] Weight loss;
[20] Somatic concerns;
[21] Lack of interest in sex.

The BDI total score is obtained by summing scores on each item. Each group has four alternatives scored as 0, 1, 2 or 3. The highest possible score is 63, because if the evaluated subject marks more than one option, the affirmation with the highest evaluation will be used to calculate the total score.

The first cut-off score of the original BDI was based on clinical evaluations of depression corresponding to the scores determined. In the 1993 edition, different cut-off scores were suggested to evaluate depression intensity: 0-9, minimum depression; 10-16, mild depression; 17-29, moderate depression; and 30-63, severe depression. However, the authors believe that the cut-off may vary according to the purpose of the examiner and the sample. Thus, if the purpose is to detect the maximum number of depressed people the cut-off should be lowered to minimize false negatives. Hence, even if the number of false positives increases, this method is useful for screening possible cases of depression.

**Statistical treatment.** Statistical program: The program used for statistical analysis was SIGMA STAT for Windows - Version 2.0 by Jandel Corporation.

*Data relating to Excessive Daytime Sleepiness by the Epworth scale* - Analyzed by the Kruskal-Wallis test with Dunn's multiple comparison, p<0.05. Data was expressed as MEDIAN (MINIMUM - MAXIMUM).

*Data relating to the evaluation of depression by the Beck Inventory* - Analyzed by the Chi-square test, with p<0.05, expressed in percentage.

## RESULTS

**Figure 1:**
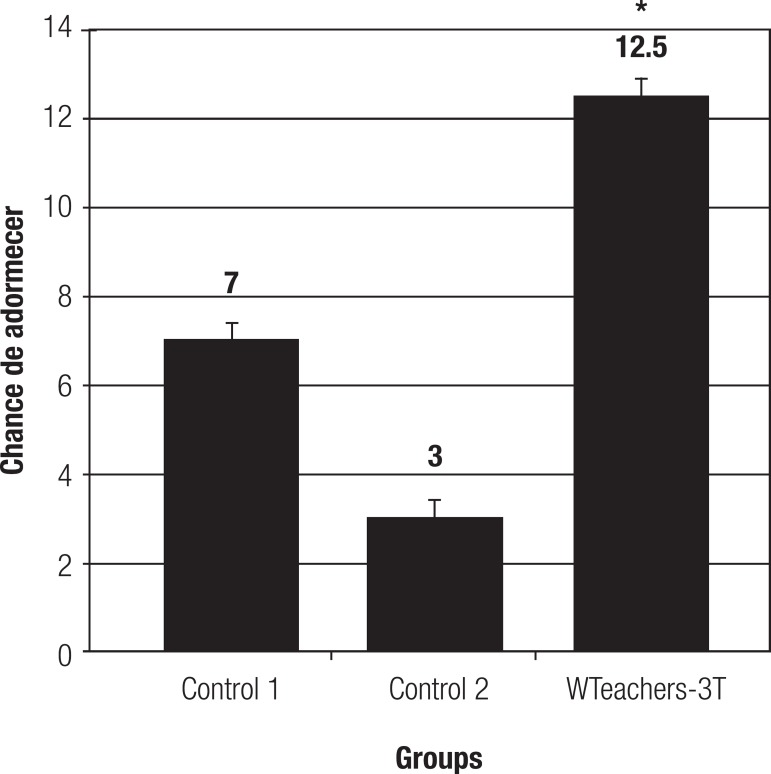
Result for presence of excessive daytime sleepiness in 201 teachers from public schools of Quipapa/PE, comprising 38 subjects working only 1 shift, 40 working 2 shifts and 123 working 3 shifts. Subjects were evaluated by self-reported questionnaire with eight questions called the Epworth Sleepiness Scale (ESS), describing in detail the chance of dozing off. Statistics: Kruskal-Wallis test with the Dunn multiple comparison test, p<0.05. Data expressed as Median (Minimum - Maximum).

**Presence of excessive daytime sleepiness-EDS.** Female teachers from public schools of Quipapa/PE working 3 shifts had excessive daytime sleepiness, considering that for the Epworth scale, the value of excessive daytime sleepiness is above a score of 9 (Figure 1).

**Presence of depression in teachers of public schools of Quipapa/PE, Brazil - BDI.** Female teachers working 3 shifts (WTeachers-3T, n=123) had a higher percentile of mild (24%) and moderate (37%) depression compared to the control groups - Control 1: mild (8%) and moderate (11%) - Control 2: mild (5%) and moderate (15%) (Table 1).

**Table 1:** Presence of depression.

Subjects	Absence			Mild			Moderate			Severe		Total
	**Freq.**	**%**		**Freq.**	**%**		**Freq.**	**%**		**Freq.**	**%**	
Control 1	31	82%		3	8%		4	11%		0	0%	38
Control 2	32	80%		2	5%		6	15%		0	0%	40
WTeachers-3T	48	39%		29	24%		46	37%		0	0%	123
Total	111			34			56			0	0	201

Female teachers working 1 shift (CONTROL 1), n=38; female teachers working 2 shifts (CONTROL 2), n=40; female teachers working 3 shifts (WTeachers-3T), n=123. Statistics: Chi-square test p<0.0001.

## DISCUSSION

This study observed that teachers from public schools of Quipapa/PE who worked 3 shifts had excessive day-time sleepiness and a greater rate of mild and moderate depression compared to teachers working 1 (one) or 2 (two) shifts.

These results corroborate the findings of Rocha & Sarriera (2006)^14^ and Moraes et al. (2011)^15^ regarding the specific aspect of sleep disorders in teachers. However, although these results are linked, it is necessary to highlight their methodological differences. While the present study involved 201 female teachers of elementary school, comprising 38 professionals working 1 shift, 40 working 2 shifts and 123 working 3 shifts, Rocha & Sarriera (2006)^14^ assessed 161 teachers from 14 graduate-level courses of various disciplines, in a sample comprising 56% female subjects and 44% male subjects. In relation to the study of Moraes et al. (2011)^15^ there was great methodological similarity with the present study, the only difference being the quantity of female teachers in the study which numbered only 121. Nevertheless, the sleep disorders evaluated and confirmed were not related to excessive daytime sleepiness, but to other disorders, including snoring loudly, pauses in breathing (apnea), waking up choking, restless sleep, hypnagogic hallucinations, unpleasant sensations in the legs, movement of limbs, speaking while asleep, sleepwalking, nightmares, feeling anxious, tachycardia, irritability and sweating hands.

Despite the few differences in the methods mentioned above, the results raise attention given the high rate of impairment in sleep quality found among Brazilian educators. The aspect of dividing groups by shifts also proved relevant in this study since, akin to a study of stress by Albuquerque et al. (2010)^16^ using the same methodological configuration, the results pointed to greater compromise in subjects working 3 shifts.

Despite a number of different phenomena studied, these morbidities (stress, depression and sleep disorders) are intimately connected on a neurophysiological level. Porsolt et al. (1977),^17^ studying animal models, proved that stress leads to depression. Depression is not only sadness but is a disease that causes neurochemical alterations. Studies in animals and humans have shown for example, the role of serotonin in emotional disorders such as depression,^18,19^ and in regulating aggressive behavior (Moeller et al., 1998; Evenden, 1999)^20,21^ and anxiety.^22,23^

It seems that the interlacing of these morbidities is connected to the functioning of serotonergic neurotransmission. A wide variety of functions of the central nervous system involve the participation of serotonin.^24^ Some of these functions are associated with pain sensitivity, learning and memory processes as well as with dietary and emotional behaviors.^24,25^

The aforementioned factors may justify the cognitive impairment connected to sleep disorders, such as attention problems, concentration and memory^26^ which, although not evaluated in this work, have been the source of constant complaints by teachers. These impairments are evidenced by poor performance at school, work and in other aspects of the subjects' lives.^27^

Regarding the relationship between sleep and memory, it is known that the transition from wakefulness to sleep tends to affect memory, since sleep renders transfer from registers of short-term to long-term memory inactive. Decoding of the material after the beginning of sleep is similarly affected, owing to lapses that hamper mnemonic strategies which do not present sufficient strength to allow evocation.^28^ Although apnea was not investigated in this study, it is possible that some of the teachers assessed had this syndrome, since excessive daytime sleepiness was observed, constituting the main symptom of the syndrome of sleep obstructive apnea, and may lead to problems in memory function.^29^

It is clear that the cause of depression in the evaluated teachers is not attributed solely to sleep disorders but a series of factors, including lack of support at the institutions, different structures of personality, non-financial recognition of the importance of a profession that prepares for all others.

These results are not common only to the teachers of the Northeast region Brazil. Stoltz & Cezar Junior (2008)^30^ carried out a study with 1368 teachers from the State Public Network of Florianópolis in Southern Brazil. From this number of teachers, 339 teachers were undergoing health treatment in general, corresponding to 24.78% that did not fully exert their functions. Of the 339 teachers, 142 were off work due to mental illness (Mental and Behavior Disorders - F00/F99 of the international code of disease - ICD 10), accounting for 41.88% of absences in the 6-month period. It was perceived that the most frequent diseases belonged to the Group of Mental Disorders, representing almost half of the total cases. Among the most frequent diagnoses of these Disorders, Mood Disorders had the highest rate, observed in 52.55%. In this group of Mood Disorders, both Depressive Episode and Moderate Depressive Episode were ranked joint top statistically, having a frequency of 15.38%.

Helplessness in a large proportion of professionals of the education sector does not seem to affect only the question of mental health, which is significant, but also the social identity of the teachers. According to Paula (2010)^31^, the image of teachers in recent years has suffered due to the lack of financial incentive and also because of the superficial training of a large proportion of teachers in the country. This evident lack of preparation of many teachers of elementary and high school level is possibly the result of lack of motivation or financial difficulties precluding them from pursuing their professional training. However, this reality is not exclusive to Brazilian schools. According to Nóvoa (1992),^32^ until the 1960s, the Portuguese government maintained an attitude of vigilance in relation to teachers' training and intensified the mechanisms of ideological control: relaxation of the pre-requisites for admission into normal teaching, reduction in the contents and time for training, implying a lower intellectual and scientific demand.

Thus, as with the situation of Luso-Brazilian teachers, other studies have shown similar realities such as the case of teachers from Hong Kong cited by Pennington (1995).^33^ According to the author, besides a high level of daily stress affecting school teachers and students, the teachers are not incentivized by favorable working conditions or opportunities for personal development, on the contrary, the educators of Hong Kong have little autonomy over the work they carry out, few opportunities for collaborative work, and a lack of incentives such as promotions or recognition by society.^31^

Of broader concern however, is the fact that these conditions are not realities confined to Brazil, Portugal or Hong Kong, but closely reflect a world stereotyped image of teachers. Zeichner (2003)^34^ affirms that in most countries, teachers earn low wages in comparison to pay levels of other professionals with the same or inferior schooling level , obliging many education professionals to engage in two or more professional activities in order to survive with the minimum comfort. Possibly, this lack of financial recognition of teachers is one of the factors affecting the image that teachers have of the profession and consequently of themselves.

In this sense, in order to remain a teacher, there seems to be the need to create a sort of psychic defense so as not to quit or fall ill, like a love of the profession. The work of the teacher, in this context, has a preponderant factor, for "the teacher, in order to perform the work in a way to reach their objectives, the establishment of the affection connection is practically mandatory".^35^

This said, the impression is that the teaching profession makes the individual fragile, that is, in the educational context it could be said that teachers undergo a continued process of building their identity by means of dialogic relationships with colleagues, coordinators, parents and mainly with their students. Thus, it is understood that teachers build or rebuild their professional identity when they interact with the people around them, when reiterating personal and social conceptions about themselves and the profession, when negotiating and renegotiating their personal-professional identity, rendering this a process of constant development. Consequently, a defining element of the identity is permanent instability.^36^

This instability seems to place teachers in a situation of having to "kill a lion" per day in order to prove their merit. This becomes tiring and places them under constant pressure. Although, depression seems to be caused by thought processes when not of genetic origin and also becomes physical in other ways. Therefore, depression cannot be conceived as a moment of sadness, but a disease that causes neurochemical disorganization.

Depression is one of the most common mental disturbances and constitutes a contemporary public health problem, with a prevalence of 2-3% in men and 5-9% in women. However, the overall risk throughout life for a major depressive episode is double this prevalence. The condition is associated with an increase in morbidity and mortality, through not only committing suicide, but also owing to increased risk or worsened prognosis of other chronic so-called "organic" diseases. Coronary disease and diabetes mellitus type 2 represent two examples of well-established consequences.^37^

According to the Diagnostic and Statistical Manual of Mental Disorders, Fourth Edition (DSM-IV), a major depressive episode (in the absence of episodes of mood elation and psychotic disorders) is defined when a sick individual experiences a depressive mood and/or loss of interest or pleasure for longer than two weeks, or for at least four more weeks in the case of manifesting both these symptoms. Signs and symptoms include the following: loss of weight or increased appetite; insomnia or hypersomnia; psychomotor retardation or agitation; fatigue or loss of energy; feeling of devaluation or disproportionate guilt; indecisiveness or loss of concentration or the capacity of thinking; suicidal ideation or suicide attempts, or constant thoughts about death.^37^

When it is said that depression is a disease affecting the neurochemistry of the nervous system this illustrates the gravity of the problem because the individual loses control of everything, that is, one cannot simply say: stop being sad and go to the church and everything will be fine. From a morphofunctional perspective, regarding the neuronal circuits of depression, it is known that different brain regions mediate the diversity of the aforementioned symptoms in relation to depression.^38^

There are three sub regions of the prefrontal cortex (PFC) most frequently implicated in depression physio-pathology, namely, the ventromedial PFC, lateral orbital region and dorsolateral region.^39^ In the ventromedial PFC, the blood flow is increased in depressed subjects showing a reduction in grey matter, with this cortex mediating in theory, the conscious aspects related to pain (physical), anxiety and depressive ruminations.^40^ The lateral orbital region, which normally suppresses or modulates emotional responses, has increased activity in major depression, maybe to compensate for the excess limbic activity. The dorsolateral PFC shows a decrease in metabolic and grey matter, where this region participates in interconnection with the portion of the dorsal anterior cingulate cortex, in cognitive paths, and is also the core of working memory. Thus, this region mediates apathy, psychomotor retardation and alterations in attention and working memory.^41^

Several clinical and preclinical studies have reported findings that support these hypotheses, i.e. a decrease in the density of serotonin (5-HT) and NA receptors in the prefrontal cortex and limbic structures, increased levels of MAO in brains of depressed subjects, decreased number of neurons both in the *locus coeruleus* and dorsal raphe nucleus and increased turnover of 5-HT in depressed individuals.^42^

The hippocampus, the most studied structure in the context of depression, is especially vulnerable to mechanisms of neurotoxicity induced by stress, due to its "central" location. It is also a mood modulator and crucial to the formation of new memories. The most common dysmorphology in major depression is a reduction in its mass, while some authors have also found changes in its segments or shape, implying a role in depression neu-robiology.^39^ This seems to confirm the participation of stress in the genesis of depression.

The fundamental response of stress consists of the corticotropin-releasing hormone (CRH) and the *locus coeruleus*-noradrenaline system (LC-NA), with the respective effector peripheral extensions: the hypotha-lamic-pituitary-adrenal axis (HPA) and the sympathetic division of the autonomic nervous system, although the latter is not contiguous, but interconnected to the *locus coeruleus*-noradrenaline system (LC-NA). These two systems influence one another mutually and positively and are also counter-regulated by the hippocampus in prefrontal cortex - both having inhibitory aspects - and by the amygdale, with a stimulatory function. It is the latter that detects and integrates the "stress information" and may trigger the adaptation response through the effector systems.^43^

A meta-analysis of the genetic studies based on families showed that the relative risk of an individual presenting MD of having an affected first degree relative is 2.8, and that the number of early cases or with multiple depressive episodes tends to rise with an increase in family aggregation of the disorder. Studies of twins with a monozygotic concordance rate of 37%, also suggest that shared genetic heritage has more significance in terms of relative risk than exposure to the same environmental factors in twins that grew up together. In conclusion, although depression is a highly inheritable disorder, with 40-60% of the risk attributed to genetic factors, if the individual has a calm life, he/she may not manifest the disease and in this sense, where stress may be the trigger for the pathology.^44^

Knowledge at the level of neurobiology of depression has evolved substantially in recent years, although this disorder has a complex pathogenesis. A paradigm has been developing from philosophical considerations to etiological considerations, based on scientific method and incorporating different approaches and hypotheses, all of which are complexly interconnected and undoubtedly incomplete.^37^

Although the explanations of science illustrate its incipient nature, showing scant progress, the state of current knowledge allows us to infer that in the case of teachers, there is an urgent need for better preparation of their managers with the objective of understanding the importance of respect, financial reward and social recognition of this professional in order to avoid incessant search to top up the family income through overloaded schedules.

It is noteworthy that according to Silva (2003),^45^ one is not born with an identity. This identity is built and involves the notion of its fixation with a process resulting from acts of linguistic creation. Thus, identity is a social and cultural instable creation, that is, it is in constant transformation. According to this author (2003, p. 9697) the identity is not an essence, nor a fact of nature or culture, neither fixed nor stable, on the contrary, it is marked by instability and by constant construction:

[...] we can say that identity is a construction, an effect, a process of production, a relation, a performative act. The identity is instable, contradictory, fragmented, unconscious and unfinished. The identity is connected to discursive and narrative structures. It is also connected to systems of representations. The identity has close connections with relations of power.

Coupled with this question, that involves the construction of a professional identity, there remains another that refers to the influence of other cultures. To what extent is multiculturalism beneficial? Often times, this influence of culture of a foreign country can reach the point of interfering in the cultural identity of teachers, developing a sort of loss or devaluation or their own cultural identity. In other words, depending on how the culture of the foreign country is presented or subtly imposed, it may influence the habits of the social or professional life of a given culture. For example, the invasion of Hollywood movies seems to lead some Brazilians to overvalue cultural aspects of the United States of America.

In accordance with this notion, regarding the attitude of teachers and students of English in relation to the American culture, an overly positive attitude and almost veneration have been observed.^46^

Critical studies for example, illustrate how English language teaching has been reported by the media as being the salvation for the poorest communities of the world, in which the English language is seen as an opportunity to improve the living conditions of its individuals, which in reality does not always occur. This reinforces a historical feeling of inferiority and a devaluation complex of colonized countries in relation to the Colony. In the face of this reality, the author reinforces the need to look critically at the interests and ideologies passed by the industry of English teaching, so the teacher is not a collaborator in fostering the inequality conditions this knowledge produces in an underprivileged society.

The situation of these teachers, who are people that deal with knowledge, seems to be a little more delicate, in the face of this bombardment of information that comes from post-modernity. It seems that this professional is left to his fate. Given the above, this poses a question: is the sensation of helplessness a factor further weakening this professional today?^47^

Few readers may be aware, but in some ways the first scholar concerned about human helplessness was Freud. Among the explored approaches in the psychoanalytic literature, two lines standout: the anthropological or phylogenetic and the mentalist or ontogenetic. In the former, the question was the passage from nature to culture and the corresponding process of "humaniza-tion". By contrast, in the mentalist or ontogenetic approach, the nature-culture passage is already assumed for the species, needing only to be carried out by each new individual. Demarcated in this plan, the theory of the subject focuses on the study of stimulants or natural mental state precursors of individual subjectivity. Therefore, what differentiates the anthropological and mentalist approaches is the fact that in the former, culture is the product or something that accompanies the formation of the subject, while in the latter, culture is assumed as the generic and peripheral environment of subjectivity. As marked as the overlaps among the approaches are, didactic separation is feasible. Of the two approaches, the mentalist is far more considered in the theory of the subject by Freud.^47^

Under the mentalist approach of the subject and more precisely the discussion of stimulants and original mental states, Freud equally proposes two theses: the first, more well-known and used is that of sexuality. In this thesis, Freud views the subject as a product of libidinal history, something moved by the demand of pleasure or by sexual stimulus.

The second thesis, referred to in the corpus of the works, presents helplessness in the origin of the subject, while it is the base organic/mental condition. Described in these terms, the subject is something permanently exposed to demands which do not have natural and efficient responses, and in the absence of these see themselves, in certain contexts, as incorporating a feeling of lack of protection. It will be this state of helplessness that will drive him through life in search of support and safety. There is no specification of the stimuli and demands that act on the origin, less still of the prediction of the type of response that it will be evoked by each cause that affects the subject. The paradigmatic illustrations of the state of human helplessness involve the cases of the initial dependence of a baby, due to the lack of motor and verbal coordination, and human impotence in the face of the suffering caused by natural forces, by fate and by other humans. It is in this helplessness state that the subject is born and lasts.

Freud maintained the primacy of the sexuality thesis in subject theory, transforming it, little by little in the way of psychoanalytic thinking. The idea of helplessness was left to the wayside throughout Freud's work, thus composing the block of unsuccessful ideas in psychoanalysis. And now helplessness comes to the fore once more.^47^

In this general sense, social helplessness of teachers seems to lead them to becoming overloaded in an attempt to survive and sustain their family, obliged to give classes in several schools or colleges to afford the basic bills. This situation seems to mean an individual is restricted from furthering their quality by say, going to the dentist periodically, taking care of their skin and hair and general health, visiting relatives and friends or prioritizing leisure.

Although this study has been carried out with the care and rigor of scientific methodology, the need to carry out a more in-depth study involving the investigation of the correlation among stress, quality of life, administration time and sleep disorders emerges. Determining the correlation among these instruments of evaluation will enable identification of the focus on comorbidity. The inventory of quality of life, for example, allows identification of whether the disarrangement that causes the sequence of comorbidities is connected to social, emotional, professional or organic health aspects of the individual assessed.^48^
